# Reduction-Induced Magnetic Behavior in LaFeO_3−δ_ Thin Films

**DOI:** 10.3390/ma17051188

**Published:** 2024-03-04

**Authors:** Nathan D. Arndt, Eitan Hershkovitz, Labdhi Shah, Kristoffer Kjærnes, Chao-Yao Yang, Purnima P. Balakrishnan, Mohammed S. Shariff, Shaun Tauro, Daniel B. Gopman, Brian J. Kirby, Alexander J. Grutter, Thomas Tybell, Honggyu Kim, Ryan F. Need

**Affiliations:** 1 Department of Materials Science and Engineering, University of Florida, Gainesville, FL 32611, USA; 2 Department of Electronic Systems, NTNU—Norwegian University of Science and Technology, 7491 Trondheim, Norway; 3 Department of Materials Science and Engineering, National Yang Ming Chiao Tung University, Hsinchu 300093, Taiwan; 4 NIST Center for Neutron Research, National Institute of Standards and Technology, Gaithersburg, MA 20899, USA; 5 Materials Science and Engineering Division, National Institute of Standards and Technology, Gaithersburg, MA 20899, USA

**Keywords:** magnetic thin films, magnetoionics, ferrites

## Abstract

The effect of oxygen reduction on the magnetic properties of LaFeO_3−δ_ (LFO) thin films was studied to better understand the viability of LFO as a candidate for magnetoionic memory. Differences in the amount of oxygen lost by LFO and its magnetic behavior were observed in nominally identical LFO films grown on substrates prepared using different common methods. In an LFO film grown on *as-received* SrTiO_3_ (STO) substrate, the original perovskite film structure was preserved following reduction, and remnant magnetization was only seen at low temperatures. In a LFO film grown on *annealed* STO, the LFO lost significantly more oxygen and the microstructure decomposed into La- and Fe-rich regions with remnant magnetization that persisted up to room temperature. These results demonstrate an ability to access multiple, distinct magnetic states via oxygen reduction in the same starting material and suggest LFO may be a suitable materials platform for nonvolatile multistate memory.

## 1. Introduction

Magnetoionics are a recently introduced approach to non-volatile magnetic memory, wherein the application of a voltage across a solid or liquid dielectric medium drives ion migration (typically, H^+^ or O^2−^) in and out of a magnetic material and induces an observable change in its properties [1,2,3,4,5,6]. Magnetoionics offer some unique advantages compared to other approaches to voltage-control of magnetism in materials [7,8,9,10]. For example, magnetoionics have shown reversible magnetic property switching throughout films many tens of nanometers thick [11,12], whereas purely electronic methods of modulating magnetism in oxides are often screened within a few unit cells of the surface [13]. The ion migration process also drives a complex composition change that can electrically dope the system to trigger electronic phase transitions [14,15], cause structural instabilities and drive crystal phase transitions [5], and produce new chemical phases [11]. Magnetoionic devices have already been built that exhibit robust cycling performance and switching speeds approaching the kHz [5,11,12,16]. Most magnetoionic devices to date have functioned by switching the coercive field, magnetization, or transition temperature of the material.

This study presents an early investigation into the viability of LaFeO_3−δ_ (LFO) thin films for magnetoionic memory. The orthoferrites are an intriguing family of candidate materials owing to their combination of fast ion transport and wide variety of magnetic properties [17,18]. LFO was chosen because it is somewhat similar in chemistry to known high-temperature ion conductors like La_1−x_Sr_x_Co_1−y_Fe_y_O_3_ (LSCFO) and Ba_1−x_Sr_x_Co_1−y_Fe_y_O_3_ (BSCFO) [17,19,20,21,22,23,24], but is magnetically better understood. LFO exhibits a well-characterized G-type antiferromagnetic (AFM) ground state with one of the highest ordering temperatures of any known perovskite oxide (T_N_ ≈ 740 K) when fully oxidized [25,26,27,28,29,30]. However, ferromagnetic (FM)-like behavior has been reported many times in LFO nanoparticles and thin films [31,32,33], which is often attributed to defect- or surface-related spin physics that cants the spins forming a canted AFM state (c-AFM) rather than a true FM or ferrimagnetic state. Yet some studies have suggested routes to stabilize LFO and other ferrites in a mixed Fe valence state (i.e., Fe^3+^/Fe^2+^) [33,34,35,36], and thereby possibly drive the system into a double-exchange FM state as seen in mixed valence manganates [37] and double perovskites [38].

In this work, LFO films were reduced using a metal getter layer and thermal anneal in vacuum as a carefully controlled means of driving the oxygen migration that would be driven electrically in a device. Inspired by work highlighting the sensitivity of LFO surfaces to substrate preparation [39] and others highlighting the important role oxide substrates as sources and sinks of oxygen in oxide ionic devices [40], three common substrate preparations were used, and then LFO films and metal layers were deposited identically, and the samples annealed simultaneously to keep the film structures and oxygen migration driving force as comparable as possible between samples. The subtle change in substrate preparation led to significant differences in (1) the extent of oxygen lost by the LFO film and (2) the magnetic behavior of the reduced LFO film. Both films exhibited key characteristics of FM behavior at low temperature, including hysteresis and remnant magnetization. The results here show an ability to access two different FM-like states from the same starting materials via oxygen reduction and suggests the possibility of using LFO for nonvolatile multistate memory [41].

## 2. Materials and Methods

### 2.1. Sample Synthesis and Annealing

A set of three identical LFO films were grown on (001)-oriented SrTiO_3_ (STO) substrates from the same wafer batch (Shinkosha, Kanagawa, Japan) but prepared using different methods commonly reported in oxide film growth literature. One substrate (labeled “as-received STO”) was only degreased with acetone and ethanol in an ultrasonic bath for 5 min then dried under nitrogen gas flow prior to growth. The second substrate (labeled “DI-rinsed STO”) received the same degreasing and drying as the first, followed by a rinse in DI water and redrying. The third and final substrate (labelled “annealed STO”) was degreased, DI-rinsed, then annealed at 950 °C for 2 h under pure oxygen flow ramping at 5 °C/min during heating and cooling. The purpose of this substrate preparation variation was to test whether the reduction process in LFO was sensitive to the substrate preparation. For brevity, the results focus on the two end-points of this series: the samples grown on “as-received STO” and “annealed STO”.

After substrate preparation, the LFO film growth, metal gettering layer deposition, and oxygen gettering anneal were all performed identically, or simultaneously where possible. Next, 20 nm-thick LFO films were grown by pulsed laser deposition using a substrate temperature of 550 °C and substrate-target distance 45 mm for all samples, with a heater temperature ramp rate of 15 °C/min for both heating and cooling. An oxygen background of 0.0025 mbar (0.25 Pa) was used during heating and deposition and 100 mbar (10 kPa) during cooling. A KrF excimer laser (λ = 248 nm) with a fluence of ~2 J/cm^2^ and 3 Hz pulse repetition rate was used to ablate material from a sintered stoichiometric LFO target. Following LFO deposition, all three samples simultaneously received a 10 nm Ta metal gettering layer deposited in an ultrahigh vacuum sputtering system followed immediately by a 1 h in situ anneal at 600 °C under vacuum (*p* < 10^−9^ mbar) to drive oxygen gettering from the LFO films. The metal deposition and oxygen gettering anneal were performed in the same vacuum system without breaking vacuum.

### 2.2. Sample Characterization Methods

The substrate surfaces were characterized before and after LFO deposition using atomic force microscopy with a Veeco Nanoscope V system (Plainview, NY, USA) under ambient conditions. Crystallinity and orientation of the bare LFO films was confirmed via X-ray diffraction (XRD) on a Bruker D8 Discover system (Billerica, MA, USA) with Cu Kα radiation. Crystallinity in the annealed Ta/LFO multilayers was subsequently measured using XRD on a Rigaku SmartLab (Tokyo, Japan) with Cu Kα radiation. Characterization of atomic structure and elemental distribution were carried out using scanning transmission electron microscopy (STEM) and energy-dispersive X-ray spectroscopy (EDS). Cross-section specimens for STEM and EDS studies were made using a FEI Helios Dualbeam Nanolab 600 (Valley City, ND, USA) focused ion beam. A final cleaning cycle of the cross-section specimens was conducted at 2 keV. High-angle annular dark-field (HAADF) imaging in STEM was performed using a Themis Z (Thermo Fisher Scientific, Waltham, MA, USA) equipped with a probe aberration corrector and a four-quadrant Super-X EDS detector. The accelerating voltage of the microscope was 200 keV and the semi convergence angle was 24 mrad. EDS elemental maps were obtained with an 80 pA beam current and a pixel dwell time of 20 μs.

Element-specific local structure and magnetic analyses were performed using X-ray absorption spectroscopy (XAS) and X-ray magnetic circular dichroism (XMCD) measurements collected at 45A2 at the Taiwan Photon Source National Synchrotron Radiation Research Center (Hsinchu City, Taiwan). XAS at Fe L_2,3_-edge was taken using a fixed circularly polarized X-ray with a magnetic field of ±1 T applied in the film plane. The X-rays were incident at an angle of 30° with respect to the film surface. Temperature was set at 77 K during the collection of XAS under a total fluorescence yield (TFY) mode. The XMCD was obtained from the difference between the XAS taken with +1 T and −1 T. More XAS measurement and analysis details can be found in the Supplementary Materials. This element-specific magnetic picture was complimented by volume-averaged magnetization measurements made in a Quantum Design MPMS3 SQUID magnetometer (San Diego, CA, USA). The diamagnetic signal from the STO substrates was subtracted from the raw data by fitting the high field (3–7 T) data at 300 K.

The multilayer structural and magnetic depth profiles were measured using polarized neutron reflectometry (PNR) collected at the NIST Center for Neutron Research on the Polarized Beam Reflectometer under a saturating field of 0.7 T at 30 K and 300 K and under a “near remanence” field of 5 mT (0.005 T) at 30 K. The raw PNR data were reduced by subtracting background scans from signals, accounting for polarization efficiencies, and correcting for the beam footprint in reductus [42]. The reduced data from each sample was then co-refined to a depth-profile model using the Refl1D software package (v0.8.16) [43,44]. The reduced data were fit to a slab-layer model of our samples defined as a scattering length density (SLD) depth profile, which can be separated into nuclear and magnetic components (nSLD and mSLD, respectively). Because only the magnetization (∝ mSLD) changes as a function of temperature, we can improve the modeling accuracy by co-refining all data sets for a given sample to a single “structural” model (nSLD) that is uniform at all temperatures while allowing only the magnitude of mSLD to vary.

## 3. Results

**Figure 1:**
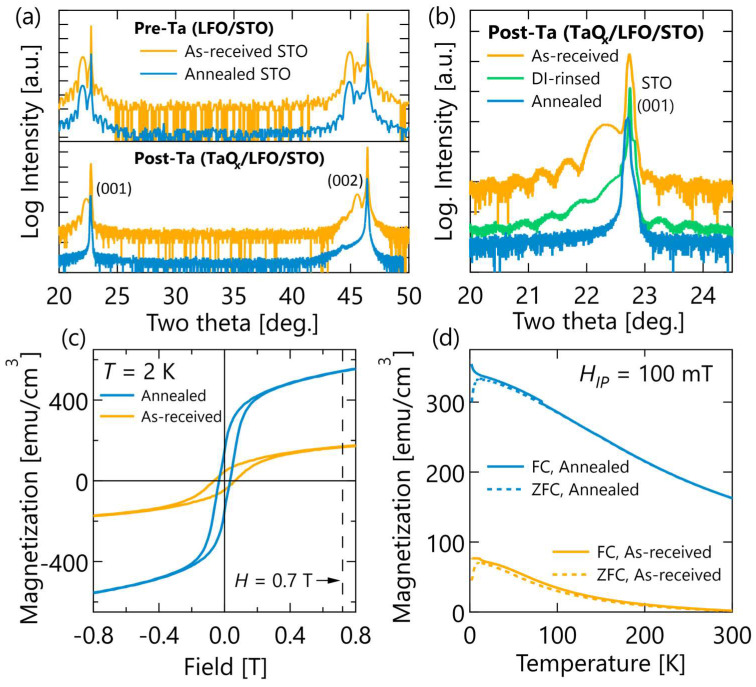
Average structure and magnetic property characterization. (**a**) Coupled 2θ-ω X-ray diffraction scans showing the (001) and (002) reflections taken before the Ta deposition (top) and after the gettering anneal (bottom). The as-received XRD data has been shifted vertically for visibility. (**b**) Comparison of the (001) reflection after Ta deposition and gettering annealing. (**c**) Field-dependent and (**d**) temperature-dependent magnetization of the samples on “as-received STO” and “annealed STO” with the applied field in the film plane. 1 emu/cm^3^ = 1 kA/m.

In Figure 1, the average crystal structures of the films are compared using XRD measurements taken before Ta deposition (i.e., on bare LFO films) and again after the Ta deposition plus in situ oxygen gettering anneal. Prior to Ta deposition, the LFO films appear identical with an out-of-plane (oop) pseudocubic lattice parameters of 4.03 Å, thicknesses of 21 nm, and finite-thickness Laue oscillations indicating smooth film surfaces. The absence of any peaks between the (001) and (002) reflections imply the LFO films are single phase and either single crystal or highly textured. After the Ta deposition and oxygen gettering anneal, the LFO on as-received STO (plotted in yellow throughout) still possessed a (001) pseudocubic reflection and Laue oscillations, indicating retention of the original perovskite structure and reasonably sharp interfaces. In this sample, the film peaks shifted towards the STO reflection following oxygen gettering, corresponding to a decreased oop lattice parameter of 3.97 Å and a 1.5% lattice contraction. By contrast, as shown in Figure 1b, the samples grown on DI-rinsed STO (green) and annealed STO (blue) substrates show clear reduction in the LFO film peak intensity and Laue oscillations, indicating a significant loss of long-range crystallinity and interface quality in these samples after oxygen reduction.

Volume-averaged magnetometry data in Figure 1c,d shows that the crystal structure difference observed in XRD following oxygen migration correlate with notable differences magnetic property differences. While as-grown LFO films on STO (001) substrates are AFM and exhibit no hysteresis or appreciable magnetization [27,45,46,47], under in-plane applied fields, both reduced LFO films exhibit FM-like hysteresis at low temperatures and saturating fields on the order of 300 mT. The sample grown on annealed STO, which had greater loss of crystallinity, also had a significantly higher remnant and saturation magnetization at low-temperature. The difference in magnetization between the two samples at low-temperature persists up to room-temperature. At room-temperature, the sample grown on annealed STO still exhibits a non-zero magnetization near remanence, while the sample grown on as-received STO magnetization becomes diamagnetic above about 150 K, indicating the LFO film magnetic signal has become smaller than the STO substrate contribution.

**Figure 2:**
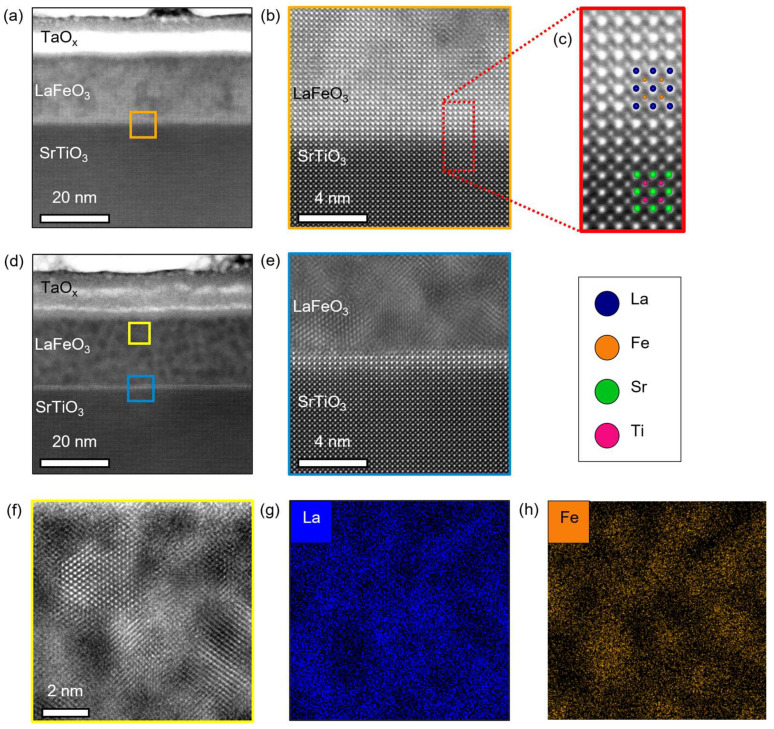
HAADF-STEM images of TaO_x_/LFO heterostructure grown on an (**a**–**c**) as-received and (**d**–**h**) annealed STO substrates. (**b**,**e**) Magnified images near the LFO/STO interface from the locations in (**a**,**d**) indicated by the orange and blue boxes, respectively. (**c**) High-magnification HAADF-STEM image from (**b**) marked by red dotted box, showing the film/substrate interface with cations overlayed. (**f**) Magnified HAADF-STEM image from the yellow box in (**d**), and corresponding EDS elemental maps of cations, (**g**) La and (**h**) Fe.

The changes in film crystallinity and magnetic properties can be better understood through a high-resolution structural analysis provided by HAADF imaging in STEM. Figure 2a–c presents HAADF-STEM images of the sample grown on as-received STO. The images in Figure 2a,b show this sample contains widespread structural defects associated with the oxygen reduction process. These are detected throughout the film and increase in density towards the Ta interface, as seen as dark image contrast in Figure 2a. Despite these defects, this sample retains a chemically abrupt, coherent interface between LFO and STO as shown in Figure 2c and large volume fractions of the perovskite structure, consistent with the retention of a film peak and Laue oscillation in XRD.

In contrast, HAADF-STEM images and EDS elemental maps from the sample on annealed STO, shown in Figure 2d–h, show a near complete loss of the original perovskite structure. This can be seen most clearly in Figure 2d,f, which highlight that the majority of the film has a nanoscale phase-separated microstructure, seen as dark inclusions within a lighter matrix. As the intensity of HAADF-STEM images is sensitive to atomic number (Z), these images imply the lighter matrix phase is La-rich (Z = 57) while the darker inclusions are Fe-rich (Z = 26). This is further confirmed by the EDS elemental maps of La and Fe shown in Figure 2g and Figure 2h, respectively. These elemental maps clearly show the segregation of La and Fe triggered by the oxygen gettering process. Despite massive chemical and structural changes throughout most of this LFO film, there remains a thin interfacial layer of perovskite LFO at the STO interface. This layer is 1.2 nm or ~3 pseudocubic unit cells thick and can be most clearly seen in Figure 2e as a bright stripe at the top of the STO substrate. Its presence intriguingly suggests some type of interfacial effect that stabilizes this region against the loss of oxygen ions. One possible explanation is the formation of a space-charge layer at the interface [48]. However, our data do not let us comment further on this phenomenon or its origin, and it is left as an open question for the community and future experiments.

**Figure 3:**
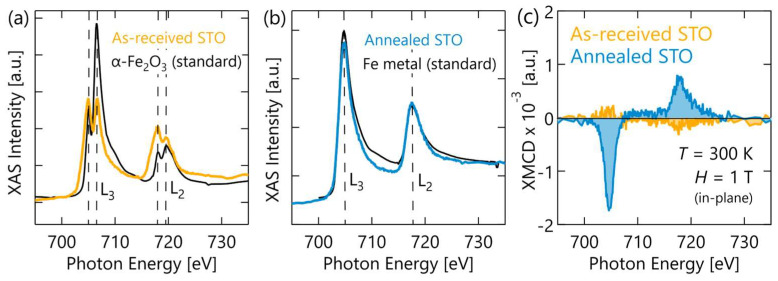
Local structural and magnetization measurements on the oxygen-gettered films. (**a**,**b**) X-ray absorption spectra compared against Fe and Fe_2_O_3_ standards. (**c**) XMCD at 300 K.

To gain a better understanding of these complex microstructures and how they connect to the magnetic behavior observed in each sample, the Fe local coordination environment and element-specific magnetism of our samples was measured using XAS and XMCD. A comparison of XAS from our samples to different Fe-valence standards is shown in Figure 3a,b. The absorption line shape of the sample on as-received STO in Figure 3a shows a split peak doublet structure, indicative of Fe ions in an oxygen ligand crystal field. The peak intensity ratio in the L_3_ doublet is known to correlate with the Fe oxidation state [49], and our nearly equivalent peak intensities indicate an average Fe valence well below the Fe^3+^ of a fully oxidized LFO film. In stark contrast, the film on annealed STO in Figure 3b shows XAS with a single peaked line shape nearly identical to that of an Fe metal standard. Similarly, the XMCD spectra from the sample on annealed STO in Figure 3c shows a line shape more consistent with pure Fe than an Fe ion in a ligand field [50,51]. These XAS measurements prove that most of the Fe in the sample grown on annealed STO is in a highly reduced local environment of Fe nearest neighbors, resembling Fe metal. This is consistent with the observation of Fe-rich nanoclusters in STEM and the greater loss of crystallinity apparent in XRD. Decomposition of LFO into La-rich and Fe-rich phases is expected when the oxygen content is reduced below a critical threshold [21,52,53].

**Figure 4:**
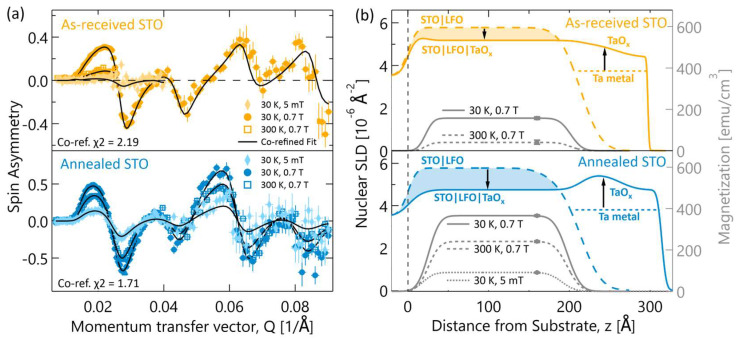
Polarized neutron reflectometry data showing a depth-resolved picture of the oxygen migration differences. (**a**) PNR data plotted as spin-asymmetry for three different temperature and field conditions. Error bars are 1σ. (**b**) The corresponding best-fit depth-profile models resulting from co-refinement of all temperature and field conditions. The nSLD of the bilayer is shown in the solid-colored lines. The dashed colored curves show the profile of bare and fully oxidized portions of each wafer (5–10% sample area). The shaded in area between these curves is proportional to the oxygen lost from each LFO film. The 5 mT mSLD curve for the as-received STO sample was nearly zero and was omitted from the plot for clarity. The point (at z = 160) on each mSLD shows the 95% confidence interval for magnetization at each temperature-field condition.

A depth-dependent picture of the magnetism and oxygen reduction in our samples was obtained using PNR measurements. By co-refining multiple PNR data sets for each sample shown in Figure 4a, structural nSLD and magnetic mSLD depth profiles were obtained for each sample. As seen in Figure 4b, each sample has two nSLD curves describing the sample’s chemical depth profile. This is because a small portion of the LFO surface (~5 mm^2^) on each sample was shadowed during Ta deposition by mounting clips and therefore did not undergo gettering-induced oxygen loss during the vacuum anneal. Since these Ta-free regions are larger than the neutron coherence length on this instrument [54], they contribute an incoherent signal to the PNR scattering and can be modeled with a distinct depth profile, plotted as the dashed curves labeled STO|LFO. These shadowed regions do not affect any of the other measurement techniques because small, cleaved pieces of the Ta-capped regions were used for those measurements.

Comparing the nSLD profiles of the Ta-capped and Ta-free regions allows for semi-quantitative analysis of the oxygen lost by LFO and gained by Ta during the gettering process. In both samples, the nSLD of the Ta-free LFO is larger than the Ta-capped LFO. Assuming only oxygen is migrating at the gettering anneal temperature, which is the case for bulk STO at these temperatures [55], then the change in nSLD directly reflects the change in average LFO oxygen stoichiometry. In this case, the filled areas between the nSLD curves are proportional to the total number of oxygen ions lost by each film. Clearly, the refined PNR profiles indicate that more oxygen was lost from the LFO film grown on annealed STO than when grown on as-received STO. This result is corroborated by the refined nSLDs in the TaO_x_ layers, in Figure 4b, and the HAADF-STEM intensity in the TaO_x_ layers, in Figure 2a,d, both of which indicate the TaO_x_ layer on annealed STO gettered more oxygen from LFO.

Several models were considered and tested to determine the best description of the magnetic depth profile in each sample. A combination of uniform magnetization profiles in the Ta-capped LFO profile and mSLD fixed to zero in the Ta-free profile were found to be the best fit to the PNR data. In agreement with our magnetometry and XMCD data, the sample grown on annealed STO was refined to have larger magnetization at all temperatures and have a non-zero magnetization at room temperature. It is important to note that the uniform magnetic profiles refined from PNR are in fact consistent with the inhomogeneities seen in STEM. This is because the inhomogeneities are several orders of magnitude *smaller* than the neutron coherence length. In this case, thousands of inhomogeneities are averaged over laterally in each scattering event. Therefore, the uniformity in the magnetic depth profiles is an indication that the distribution of *magnetic* inhomogeneities in LFO are approximately uniform along the growth direction, which agrees reasonably well with the STEM images in Figure 2.

## 4. Discussion

The data presented here from multiple complimentary techniques create a clear and consistent picture of the differences between our samples’ microstructure and oxygen reduction. Specifically, our results show that the LFO film grown on annealed STO underwent greater oxygen reduction than the film on the as-received STO substrate. This is most strongly supported by the PNR depth-profile refinements showing greater change in the LFO and Ta layer compositions after oxygen migration, but it is also supported by the greater loss of crystallinity and more reduced Fe valence state observed in our XRD and XAS measurements. As a result of this greater reduction, that LFO film underwent phase segregated into metal Fe nanoclusters surrounded by a disordered, La-rich matrix as confirmed by XAS and STEM-EDS. The presence of Fe metal and likely other Fe-rich decomposition products (e.g., Fe_3_O_4_, Fe_2_O_3_) in this sample can explain the more robust FM-like behavior. In contrast, the LFO film grown on as-received STO lost less oxygen during the gettering anneal and consequently maintained a large volume fraction of the initial perovskite structure. This is proven by the presence of a film diffraction peak in XRD and multiplet splitting of the XAS signal. The smaller magnetization values in this sample, and the fact that magnetic hysteresis and remnant magnetization only appear at low temperature, are consistent with previous reports of point-defect-induced canting of the parent G-type AFM structures and suggest similar physics here [32,33,34]. The complexity of the reduced LFO film microstructures, coupled with the dependence of Fe and Fe oxide magnetic properties on nanoparticle dimensions and interfaces, makes a deeper analysis of the magnetic ground states challenging. Towards that end, a brief comparison of in-plane and out-of-plane magnetic behavior is provided in the Supplementary Materials, but future studies that include micromagnetic modeling are needed to fully appreciate how the magnetic properties observed here are derived from these structures.

While it is clear that different oxygen reduction occurred and led to the formation of different microstructures and magnetic properties, the question of why different substrate preparations caused these differences cannot be answered by the methods used here. One possibility suggested in the literature is a difference in the width of surface terraces between the substrates. Wider terraces have been suggested to create a lower energy barrier for oxygen ion migration across the film/substrate interface [39]. Within this hypothesis, one expects films grown on substrates with wider terraces to undergo less net oxygen reduction during gettering because faster ion transport across the substrate interface can better replenish oxygen lost by the film to the metal capping layer. AFM measurements of the STO substrate surfaces before and after pre-growth treatments (see Supplementary Materials) show the annealed STO substrate had wider terraces before and after annealing than the other samples. However, the measurements presented here show that LFO grown on annealed STO underwent greater net oxygen reduction and conflict with this surface-terrace-based hypothesis. An alternative hypothesis is that annealing STO in oxygen-rich environments increases the oxygen concentration in the first few nanometers of the substrate and thereby causes a sharp drop in oxygen diffusivity in this region since oxygen diffusion is vacancy-mediated in STO and most perovskites [55,56,57]. This hypothesis agrees better with data presented here, in particular the greater reduction observed in LFO grown on annealed STO and the presence of oxygen-rich layers at the LFO/STO interface in both samples seen by STEM.

Although the reason remains to be determined, a key and clear result from this work is that LFO films can be driven via oxygen-migration and reduction to at least two magnetic ground states with different FM-like behavior. Materials that exhibit multiple unique magnetic states are current sought after for multi-state memory, where memory can be stored in significantly greater areal bit density than current binary memory technologies. Thus, the results here raise the question of whether LFO could be a platform for multi-state, magnetoionic memory. However, the results also suggest that the reduction process is extremely sensitive, and even small differences in the substrate preparation that are sometimes overlooked in experimental design can change the amount of oxygen lost from overlying films. As such, this work highlights the importance of considering the sink/source effects of substrates and capping layer in magnetoionic and other ionic-electronic devices.

## 5. Conclusions

In summary, this work characterizes the magnetic behavior and microstructure in a set of oxygen-reduced LFO films to provide an initial evaluation of their promise for magnetoionic applications. The LFO films were grown on differently prepared STO substrates but received otherwise identical treatment. Despite the large similarity between the samples, the results here show that significantly different amounts of oxygen migration occurred for films grown on differently prepared substrates. In the less reduced LFO film, grown on as-received STO, the original perovskite film structure was still largely intact, and magnetic hysteresis was only seen at low temperatures, which is suggestive of a c-AFM state. In the more reduced LFO film, grown on annealed STO, the LFO microstructure decomposed into La- and Fe-rich regions, and FM-like magnetic behavior persisted up to room temperature. These results show that multiple magnetic ground states and microstructures can be readily accessed via oxygen reduction starting from the same material. If this behavior can be precisely controlled and reversed, it will be well-suited for multistate magnetic memory.

## Data Availability

The data that support the findings of this study are available from the corresponding author upon reasonable request.

## Supplementary Materials

The following supporting information can be downloaded at: https://www.mdpi.com/article/10.3390/ma17051188/s1, a comparison of SrTiO_3_ substrate surfaces following different surface preparation methods, and additional data and discussion regarding the magnetic microstructures of reduced LaFeO_3_ films [55,58,59,60,61,62].
