# Supplementary material for: Reduction-Induced Magnetic Behavior in LaFeO3−δ Thin Films
Source: Materials (Basel). 2024 Mar 4;17(5):1188. doi: 10.3390/ma17051188 (PMC10934291; doi:10.3390/ma17051188)
Supplement: Supplementary file 1 [file materials-17-01188-s001.zip › materials-2879335-supplementary.pdf]

Supplementary Material:
Reduction-induced magnetic behavior in $LaFeO_{3-\delta}$ thin films

Nathan D. Arndt,[1,†] Eitan Hershkovitz,[1,†] Labdhi Shah,[1] Kristoffer Kjærnes,[2] Chao-Yao Yang,[3] Purnima P. Balakrishnan,[4] Mohammed S. Shariff,[1] Shaun Tauro,[1] Daniel B. Gopman,[5] Brian J. Kirby,[4] Alexander J. Grutter,[4] Thomas Tybell,[2] Honggyu Kim,[1] and Ryan F. Need[1,*]

[1] Department of Materials Science and Engineering, University of Florida, Gainesville, Florida 32611, USA
[2] Department of Electronic Systems, NTNU - Norwegian University of Science and Technology, 7491 Trondheim, Norway
[3] Department of Materials Science and Engineering, National Yang Ming Chiao Tung University, Hsinchu 300093, Taiwan
[4] NIST Center for Neutron Research, National Institute of Standards and Technology, Gaithersburg, Maryland 200899, USA
[5] Materials Science and Engineering Division, National Institute of Standards and Technology, Gaithersburg, Maryland 20899, USA
* Correspondence: rneed@ufl.edu
† These authors contributed equally to this work.

I. Sample preparation pathways

This work focuses on the reduction behavior in two $LaFeO_3$ (LFO) films grown identically on $SrTiO_3$ (STO) 001-oriented substrates that were prepared differently prior to LFO growth. Figure S1 shows a schematic visually laying out from left to right the process flow for the two main samples grown on "as-received STO" and "annealed STO". The key difference is that one substrate was annealed in an oxygen-rich environment at 950°C, which is above the temperature required for STO to oxygen exchange and equilibrate with a gas [1]. This causes oxygen to diffuse into the STO substrate filling oxygen vacancies near the surface, and cooling down through the critical temperature under O_2 traps this state and minimizes oxygen vacancies in the STO surface prior to LFO growth. Because oxygen diffusion is vacancy-mediated in STO [1], minimizing oxygen vacancies in the annealed STO substrate surface is expected to result in a smaller oxygen diffusion coefficient at the LFO/STO interface and change how much the substrate's sink of oxygen plays in the reduction of the overlying LFO.

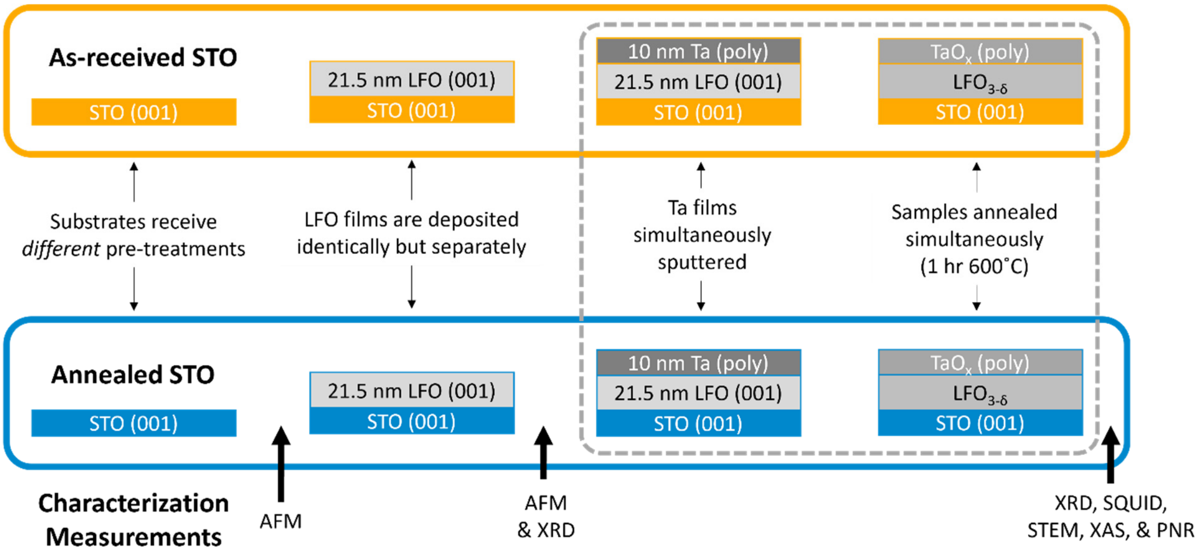

Figure S1: Schematic showing the sample processing steps in order from left to right. The steps inside the grey dashed line were done together and simultaneously.

II. Atomic force microscopy of STO substrates

a. Effects of substrate annealing on terrace width

Atomic force microscopy (AFM) was used to characterize STO substrate surfaces before and after their different preparation processes. Figure S2 displays AFM images from a control substrate, taken from the same wafer batch as the substrates used for the samples presented in the main text. The terrace widths are clearly very similar in all three images showing that negligible terrace broadening is caused by annealing at 950˚C in O_2. Instead, the main effect of the anneal that can be observed with AFM is the removal of the small, bright particulate seen in the two scans taken prior to annealing. This particulate is presumed to be $Sr(OH)_2$ formed from SrO that had segregated to the surface in the time since the manufacture's acid etch and was hydrated during the DI rinse, and it's removal by annealing at high-temperature in an oxygen-rich environment is consistent with previous studies [2,3].

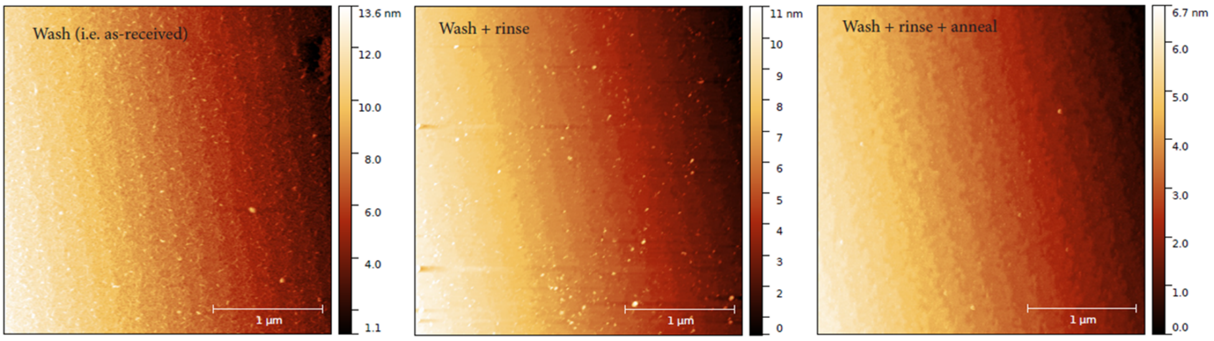

Figure S2: Atomic force micrographs on the same control substrate after solvent degreasing ("as-received"), DI rinsing/etching, and high-temperature annealing. Length scale bars are all 1 µm.

b. Surface topography before and after $LaFeO_3$ growth

Figure S3 shows the surface of the two samples discussed in the main text before (top) and after (bottom) $LaFeO_3$ (LFO) film growth. Figures S3(a,b) are the as-received STO sample and Figure S3(c,d) are the annealed STO sample. The LFO surfaces retain a similar terrace morphology as the substrate had prior to film deposition, indicating that layer-by-layer growth dominated over an island nucleation, which is consistent with RHEED oscillations observed during growth.

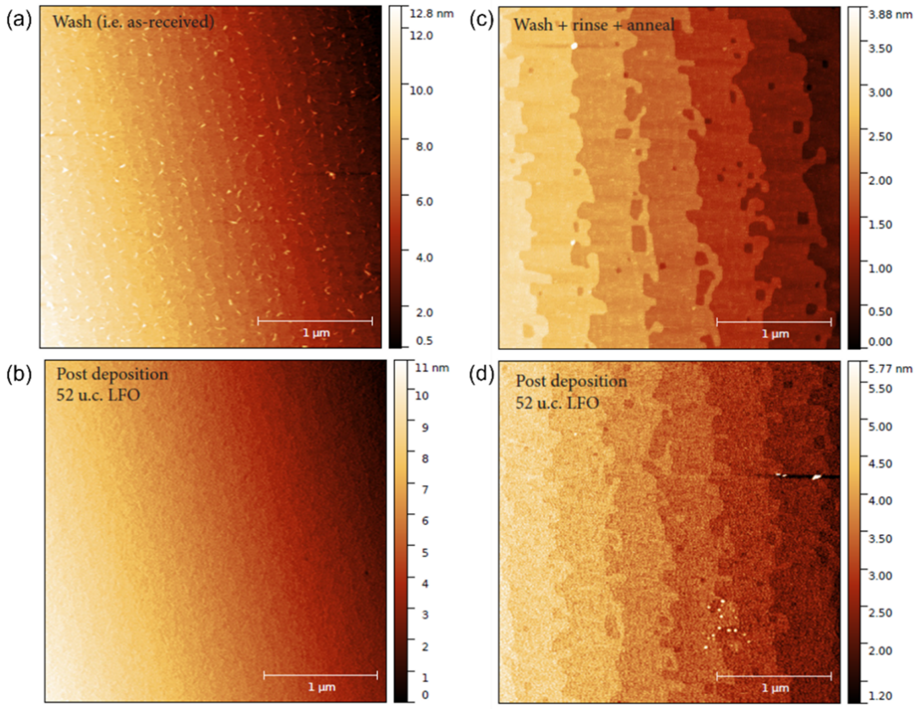

Figure S3: Atomic force micrographs from the sample on (a,b) "as-received STO" and (c,d) "annealed STO" from the main text before LFO growth (a,c) and after LFO growth but before Ta deposition (b,d).

III. Magnetic measurement data processing and anisotropy

Figure S4 shows the magnetization of the two TaOx/LFO/STO samples after the vacuum anneal that drives reduction of the LFO films. The primary contribution to the magnetic signal comes from the diamagnetic STO substrate, leading a large negative slope to the data seen in Fig. S4(a).

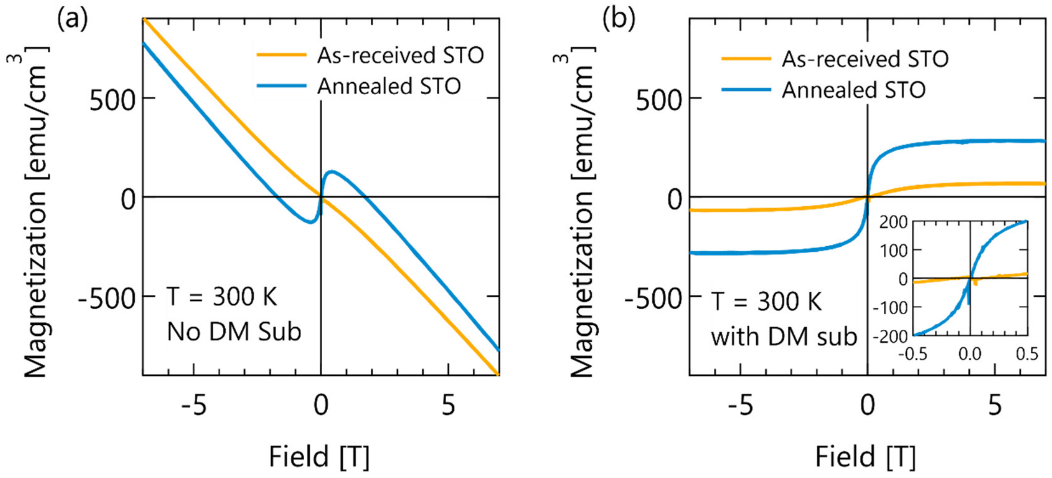

Figure S4: SQUID magnetometry data with in-plane field of the two main samples taken after LFO film reduction shown (a) without and (b) with the diamagnetic signal from the substrate subtracted. Inset in (b) shows a close-up near zero field.

After subtracting the diamagnetic signal, it can be seen that the LFO film grown on as-received STO exhibits weak paramagnetism at 300 K while the LFO film grown on annealed STO exhibits superparamagnetism or weak ferromagnetism at the same temperature. These results are consistent with the STEM images showing Fe-rich clusters on the order of 2-4 nm in diameter in the LFO film on annealed STO. Pure Fe nanoparticles typically exhibit superparamagnetic behavior or single-domain ferromagnetism with very small coercive fields (<100 Oe = 0.001 T) [4,5]

To aid the reader in interpreting the saturated magnetization values at low temperature, the 2 K in-plane field data from Fig. 1(c) of the main text has been replotted for a wider range of fields in Fig. S5 and converted to unit of Bohr magnetron per Fe. We note that the saturated magnetizations are well below the theoretical maximum moments for Fe^{3+} and Fe^{2+} of 5.9 and 4.9 μ_B/Fe, respectively [6]. However, caution should be used in interpreting the value of the magnetic saturation given the inhomogeneous microstructures and local Fe environment in both LFO films.

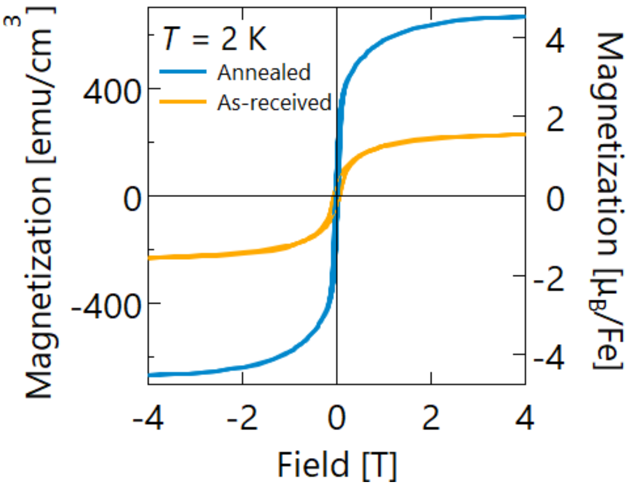

Figure S5: SQUID magnetometry data with in-plane field of the two main samples taken after LFO film reduction showing the magnetic saturation plateaux. Note: Same data as main text Fig. 1(c) plotted for a wider range of field.

Figure S6 compares the samples' magnetization at 2 K collected by SQUID magnetometry with in-plane and out-of-plane field geometries benchmarked against the 300 K in-plane data for scale. Notably, we see both samples exhibit ferromagnetic behavior with characteristic hysteresis loops at low temperature, indicating that the parent AFM structure has been disrupted in both samples.

The LFO sample grown on as-received STO shows a greater difference in coercive field anisotropy, with values of 160 Oe (0.016 T) and 650 Oe (0.065 T) for out-of-plane and in-plane, respectively. By contrast, the LFO sample on annealed STO shows similar coercivities with values of out-of-plane and in-plane fields of 325 Oe (0.033 T) and 265 (0.027 T), respectively. This difference in coercive field anisotropy is likely due to the Fe in LFO film on as-received STO (in an oxygen ligand field) having larger magnetocrystalline anisotropy than the Fe in the LFO film on annealed STO (in metal-like, Fe-rich nanoclusters). The saturation magnetization of LFO film on annealed STO is ~3x larger than the LFO film on as-received STO for both field geometries. This is consistent with the presence of ferromagnetic and/or superparamagnetic Fe-rich clusters in the sample on annealed STO.

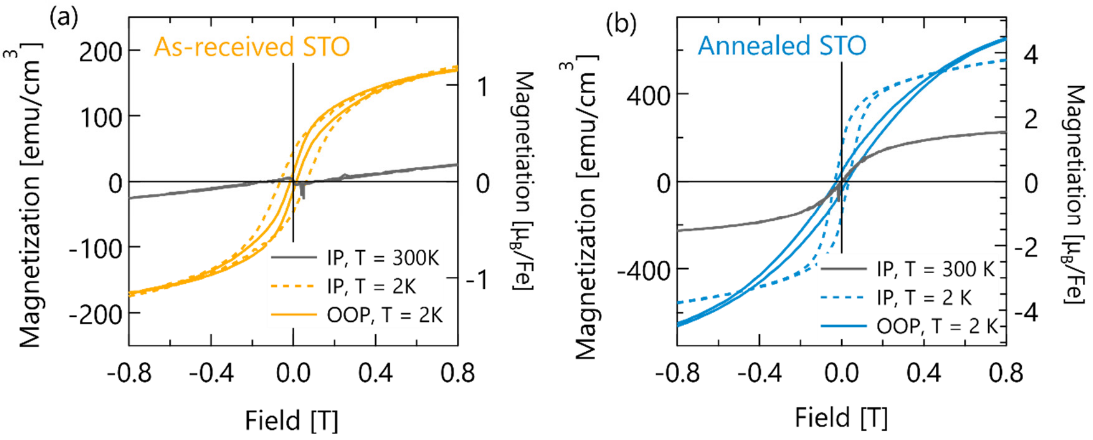

Figure S6: Direction- and field-dependent SQUID magnetometry measurements of the oxygen-gettered films on an (a) as-received STO and (b) annealed STO substrate.

IV. Elemental analysis of the LFO/STO interface

Scanning transmission electron microscopy and energy dispersive spectroscopy (STEM-EDS) were used to collect a semiquantitative picture of elemental variation across the LFO/STO interface for the sample grown on annealed STO collected after the oxygen gettering anneal in which the Ta cap reduces the LFO film. Figure S7 shows this data, where it can be seen that the oxygen concentration in the LFO film is lower than the STO substrate.

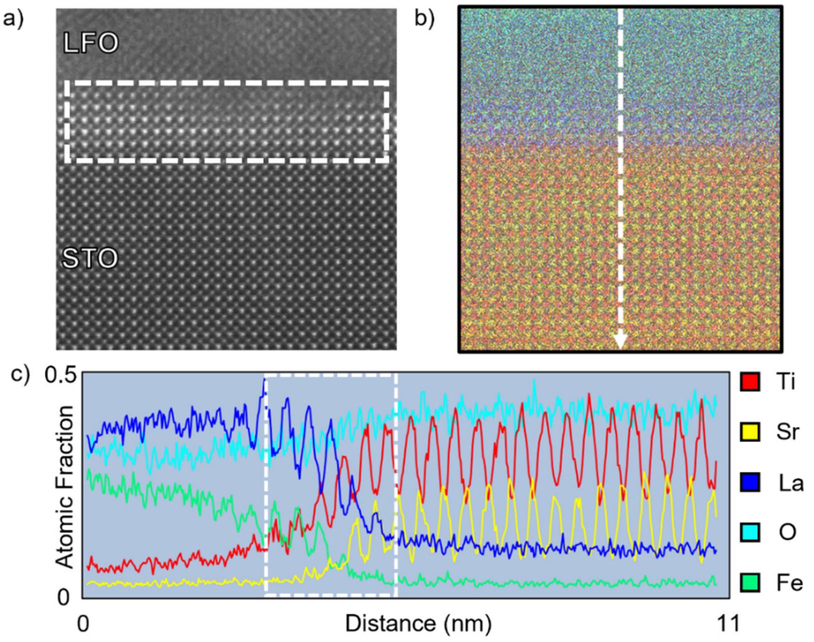

Figure S7: (a) Atomic resolution HAADF-STEM image of the LFO/STO interface for sample grown on annealed STO. (b) corresponding EDS map with the dashed white line indicating the direction of the line profile shown in (c). The dashed white box in (c) corresponds to the area in the dashed white box in (a).

V. Magnetic depth profile refinement method

The raw PNR data were first reduced by subtracting background scans from the signal, adjusting for polarization efficiencies, and correcting for the footprint of the incident beam. Numerical refinements of the reduced PNR data were then done using a combination of two algorithms executed in the Refl1D software package. First, because of the large dimensionality of the parameter space in reflectometry measurements (n ~ 20), the DREAM algorithm was used to rapidly sample a wide parameter phase space and identify the general parameter space with the best fits. DREAM is a Markov chain Monte Carlo method with a differential evolution step generator that explores parameter space using a random walk similar to simulated annealing algorithms, always accepting a better point in phase space but also accepting a worse point depending on how much worse and at what point in the regression it is. Second, a Nelder-Mead simplex algorithm was used to find the absolute minimum within the region of phase space identified by the DREAM algorithm. The Nelder-Mead method, also sometimes referred to as the amoeba or polytope method, is a "downhill" algorithm that creates a simplex of $n+1$ points, where n is the dimensionality of the parameter space, then continually moves the simplex point where the goodness of fit is greatest eventually converging on the local, and hopefully global, minimum. Error on refined parameters (e.g., layer thicknesses, magnetization) can be reported as XX% confidence intervals (CI) determined by refining a final time using the DREAM algorithm and starting from the downhill optimized parameters, where the XX% CI proportional to the interval range that contains XX% of the accepted hops.

The above refinement procedure was applied to different "slab" models of the film heterostructure, wherein the TaOx/LFO/STO films are broken into at least one slab per composition and the substrate treated in a semi-infinite approximation. Each slab has a nuclear scattering length density (nSLD), thickness, and roughness. Each layer can also have a magnetic scattering length density, but that parameter can be fixed to zero if known or desired to have no net magnetization, as was done for the TaOx slabs. The interfacial roughness between slab layers was modeled using the Nevot-Croce approximation, which treats the roughness as a Gaussian distribution. Roughness from each interface is propagated throughout the entire sample stack as described in Ref. [3] and implemented in the latest version of Refl1D. Many different slab models were compared for each sample to determine the "best" model, taken here to be the one with the combination of fewest slabs (fitting parameters) and best goodness-of-fit, here calculated as a reduced χ^2 value from the co-refinement of multiple temperature and field PNR datasets from the same sample. The structural and magnetic depth profiles plotted in the main text correspond to models in which the reduced LFO layer is split into three slabs, thin non-magnetic layers (~2 nm) at each interface and a thick magnetic interior layer (~19 nm). In the Ta-free regions of the LFO film, that were shadowed by clips during Ta sputtering, the interior LFO layer was forced to be non-magnetic by setting its mSLD parameter to zero.

References

1. De Souza, R.A. Oxygen Diffusion in $SrTiO_3$ and Related Perovskite Oxides. *Adv. Funct. Mater.* **2015**, *25*, 6326–6342. https://doi.org/10.1002/adfm.201500827.
2. Connell, J.G.; Isaac, B.J.; Ekanayake, G.B.; Strachan, D.R.; Seo, S.S.A. Preparation of Atomically Flat $SrTiO_3$ Surfaces Using a Deionized-Water Leaching and Thermal Annealing Procedure. *Appl. Phys. Lett.* **2012**, *101*, 251607. https://doi.org/10.1063/1.4773052.

3. Raisch, C.; Chassé, T.; Langheinrich, Ch.; Chassé, A. Preparation and Investigation of the A-Site and B-Site Terminated SrTiO3(001) Surface: A Combined Experimental and Theoretical x-Ray Photoelectron Diffraction Study. *Journal of Applied Physics* **2012**, *112*, 073505. https://doi.org/10.1063/1.4757283.
4. Papaefthymiou, G.C. Nanoparticle Magnetism. *Nano Today* **2009**, *4*, 438–447. https://doi.org/10.1016/j.nantod.2009.08.006.
5. Carvell, J.; Ayieta, E.; Gavrin, A.; Cheng, R.; Shah, V.R.; Sokol, P. Magnetic Properties of Iron Nanoparticle. *Journal of Applied Physics* **2010**, *107*, 103913. https://doi.org/10.1063/1.3428415.
6. Mugiraneza, S.; Hallas, A.M. Tutorial: A Beginner’s Guide to Interpreting Magnetic Susceptibility Data with the Curie-Weiss Law. *Commun Phys* **2022**, *5*, 95. https://doi.org/10.1038/s42005-022-00853-y.
